# Primary Malignant Melanoma of Maxilla: Report of a Case with Discussion

**DOI:** 10.1155/2014/624306

**Published:** 2014-11-17

**Authors:** G. Shirisha Rani, T. Vinay Kumar, Balaram Kolasani, Md Rezwana Begum, Anu Priya Srinivasan

**Affiliations:** ^1^Department of Oral Medicine & Radiology, Meghna Institute of Dental Sciences, Varni Road, Mallaram, Nizamabad 503003, India; ^2^Sri Padmavathi Hospital, Medak, Narayankhed 502286, India; ^3^Government Dental College & Hospital, Gunadala, Vijayawada 520008, India; ^4^Department of Oral Medicine & Radiology, Githam Dental College, Gandhinagar Campus, Rushikonda, Visakhapatnam 530045, India; ^5^Department of Oral Pathology, AECS Maaruti Dental College, Kammanahalli, Bangalore 560076, India

## Abstract

Primary oral malignant melanoma, very rare neoplasm of melanocytic origin, usually presents as a bluish black to tan-brown colored lesion Which is accounting for 0.2 to 8% of all melanomas, 1.6% of all head and neck malignancies, and 0.5% of all oral neoplasia. In general, the prognosis of oral melanoma is poor and worse than that of cutaneous melanoma. Here a case of oral malignant melanoma is presented, which was undetected during the first visit to a dental clinic. When a simple oral surgical treatment was carried out in that region, it resulted in the appearance of a massive pigmented lesion which was histopathologically diagnosed as malignant melanoma. This paper is presented to reemphasize the fact that any pigmented lesion in the oral cavity should be viewed with suspicion and proper investigation (biopsy) should be carried out to rule out any untoward experiences later.

## 1. Introduction

Primary malignant melanoma of the oral cavity (POM) is an infrequent neoplasia of very aggressive characteristics originating from the malignant transformation of the melanocytes of the mucosa [1]. Presence of POM has been described virtually in all sites and organ systems into which neural crest cells migrate [2, 3]. The World Health Organization (WHO) has defined mucosal malignant melanoma as a malignant neoplasm of melanocytes or of melanocyte precursors [1, 4]. It is characterized by the proliferation of atypical melanocytes at the epithelial-connective tissue interface, associated with upward migration into the epithelium and by invasion of the underlying connective tissues [1].

Over 90% of melanomas occur on the skin. Primary malignant melanoma of the head and neck is a rare tumor, accounting for less than 1% of all melanomas. Half of such melanomas occur in the oral cavity, followed by the nasal cavity (44%) and sinuses (8%). In the oral cavity, the most frequent sites of occurrence are the hard palate and the maxillary gingiva [2].

Oral malignant melanomas demonstrate significant heterogeneity in morphological features, developmental process, and biological behaviour. Hence oral malignant melanoma still represents a diagnostic challenge [5].

Melanoma is a major health problem. When discovered early and fully excised, melanoma is highly curable. However, once metastatic disease develops, treatment options are limited and survival is generally measured in months. Patients with stage III melanoma (involvement of regional lymph nodes) have a 5-year survival of approximately 50% [2].

## 2. Case Report 

**Figure 1:**
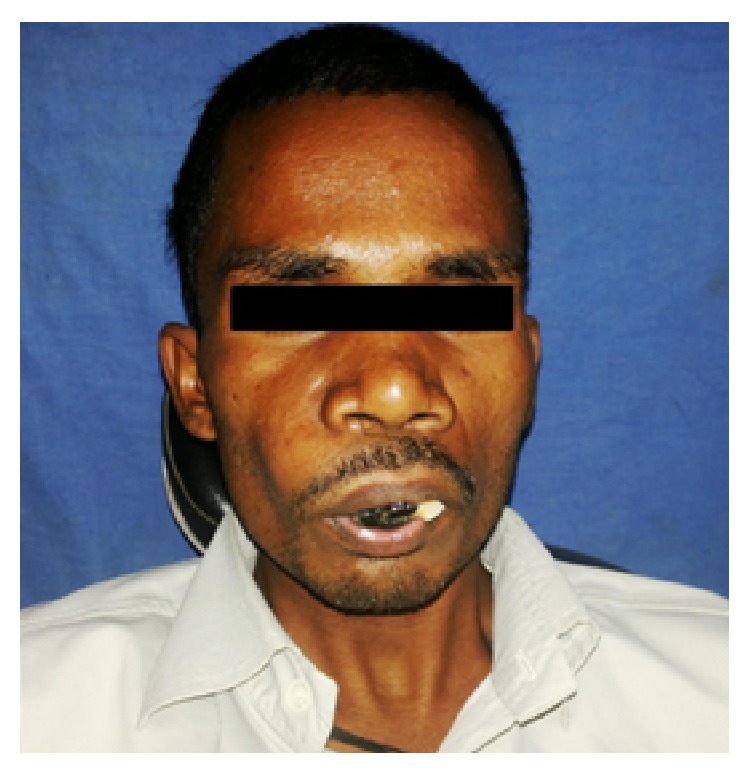
Incompetent lips with blackish growth.

**Figure 2:**
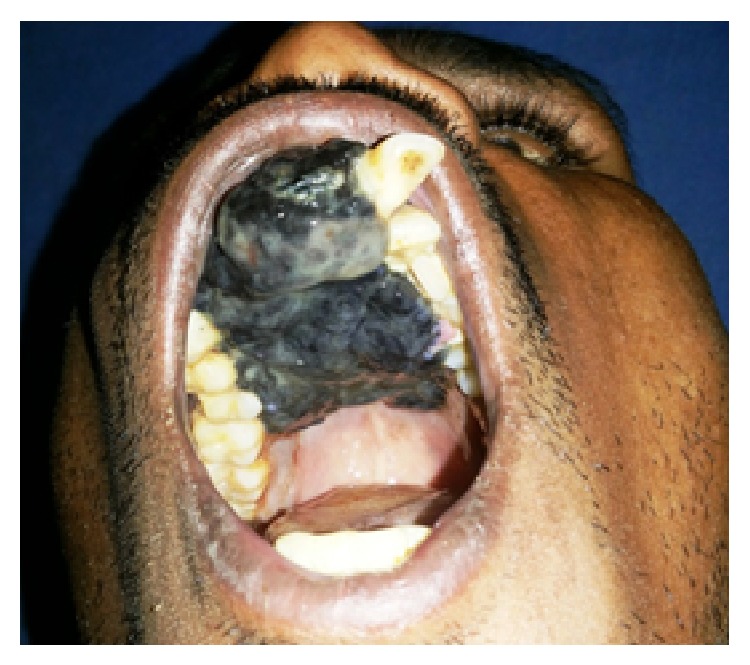
Exophytic growth covered entire hard palate.

**Figure 3:**
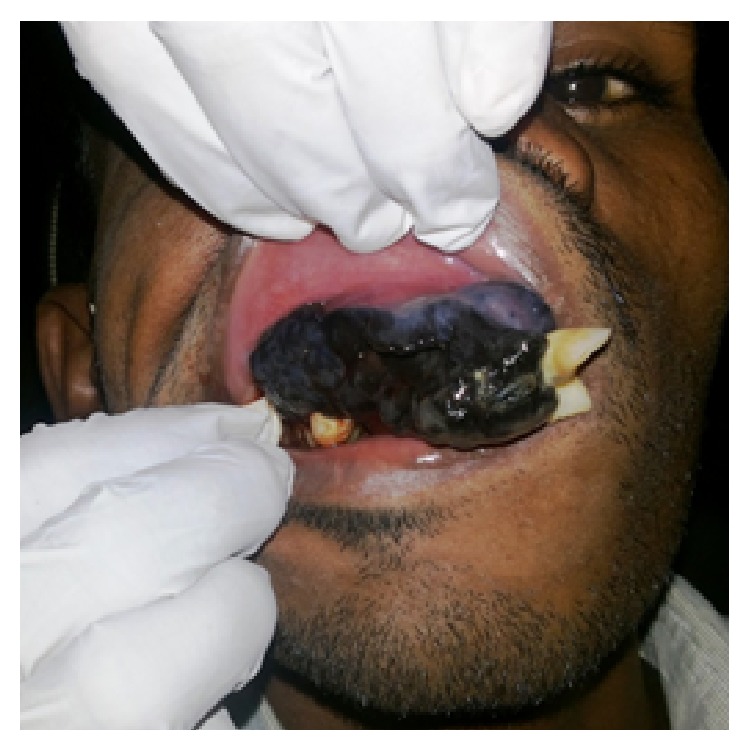
Blackish growth involving entire maxillary anterior alveolus with displacement of teeth.

25-year-old male patient presented with a complaint of black colored growth on the anterior maxilla since 3 months. Patient gave the history of extraction of loosened upper front tooth which was associated with a black tinted peanut sized growth. Following extraction the growth gradually increased and attained to the present size with change of colour to dark black. Growth was associated with difficulty in eating, speaking, loosening of adjacent teeth, mild pain, and frequent bleeding on provocation. Patient gave history of no such similar disorders in the family, and no adverse habits. The patient was moderately built and nourished, and vital signs were normal. General examination did not reveal any other similar findings. Extra oral examination shows incompetent lips with obliteration of nasolabial fold on right side (Figure 1). Lymph nodes were nonpalpable. Intraoral examination showed grayish black colored exophytic growth on maxilla involving maxillary gingiva and hard palate (Figures 2 and 3) of approximately 7.5 × 5.0 cm^2^ size extending anteriorly on gingiva from 16 to 24 by crossing midline with displacement of 11, 21, and 22 and superiorly to depth of maxillary anterior vestibule, and posteriorly to soft palate, involving whole of the hard palate with protrusion into oral cavity. Tooth 12 was missing. Surface was irregular, shiny with teeth indentation at the borders. On palpation the lesion was slightly tender, soft in consistency, smooth textured, and fixed to underlying structures and bleeds on probing. Based on the clinical findings provisional diagnosis of oral malignant melanoma involving maxillary alveolus, palate was given. Differential diagnosis of any vascular related disorders was suspected.

To rule out the diagnosis, primary investigations such as aspiration, haematological and urine examinations, incisional biopsy, and CT were done.

**Figure 4:**
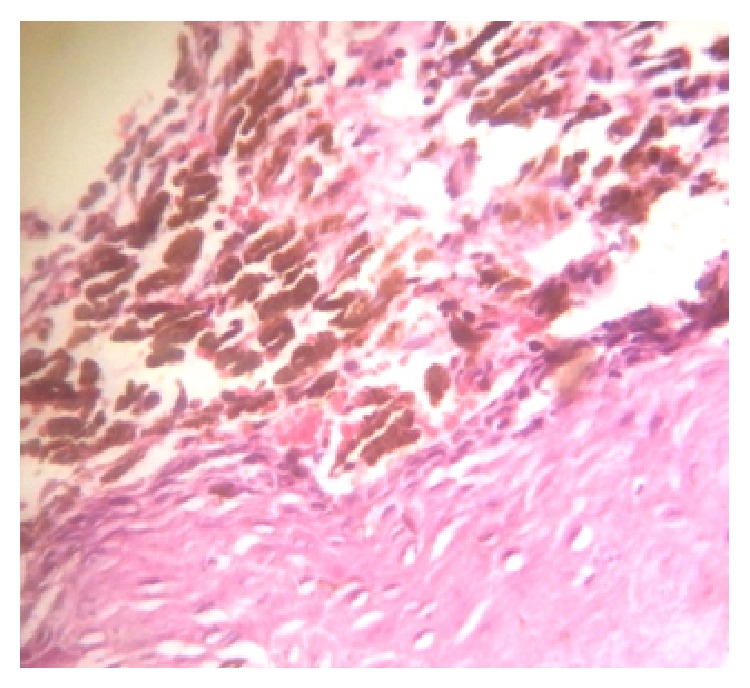
Strips of squamous epithelium with proliferation of tumor cells arising from basal layer infiltrating deeply into the stoma. Individual cells are round to spindle with melanin pigment.

Aspiration was negative. Haematological, urine examinations did not reveal any significant findings, which showed that the lesion was nonhematological. Histopathology revealed strips of squamous epithelium with proliferation of tumor cells arising from basal layer infiltrating deeply into the stoma. Individual cells are round to spindle with melanin pigment. Nuclei are binucleated and large mononucleated giant cells are seen, which is suggestive of malignant melanoma (Figure 4).

**Figure 5:**
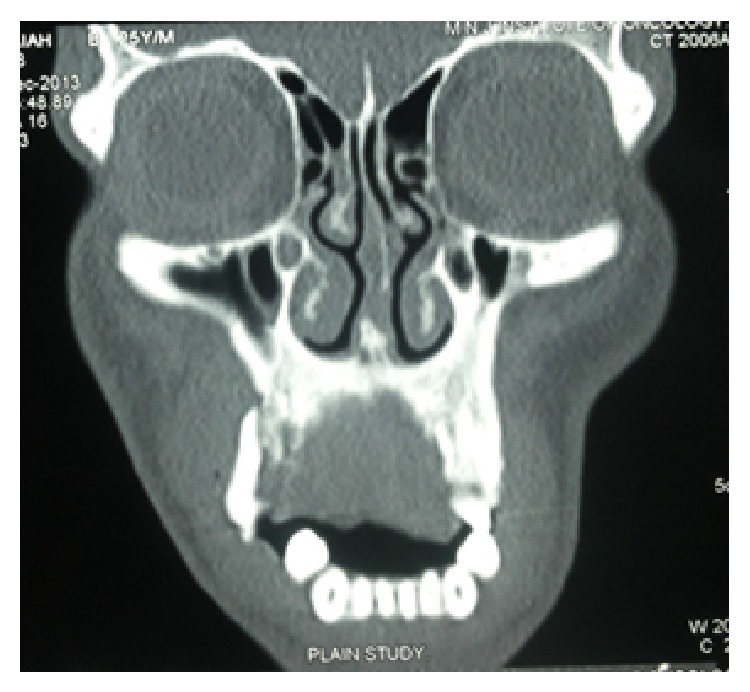
Coronal CT scan showing ill-defined enhancing soft tissue mass on palate with bone destruction.

**Figure 6:**
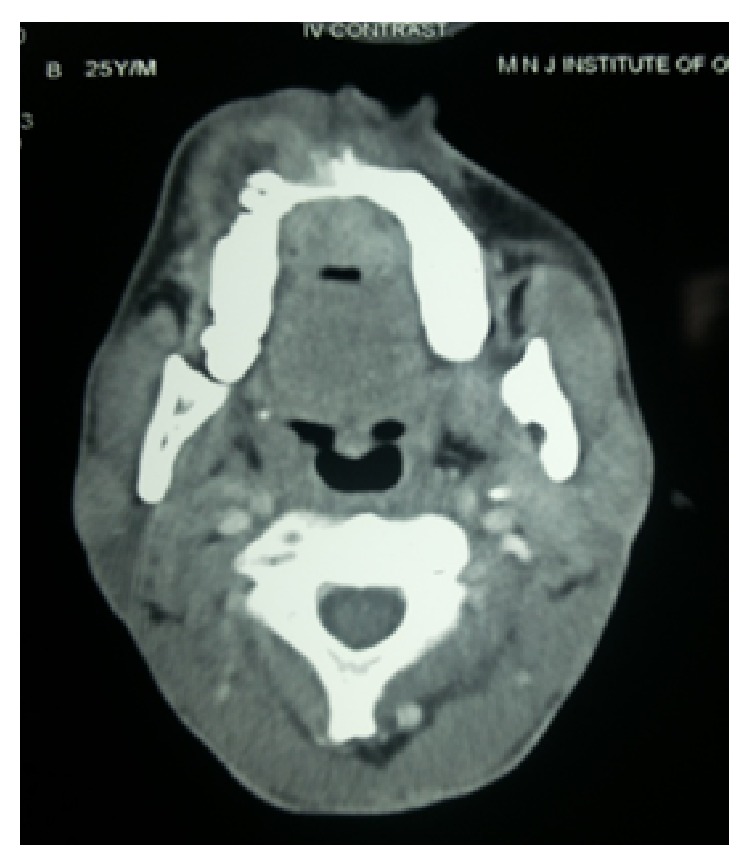
Axial CT scan showing ill-defined enhancing soft tissue mass on anterior hard palate extending posteriorly up to the soft palate on right side.

CT revealed a soft tissue density mass in the maxillary alveolus, hard palate protruding into oral cavity measuring 5.4 to 3.2 cms (Figures 5 and 6). The mass is eroding the alveolus in the midline and eroding free molars. And also causing displacement of anterior teeth. Mass does not involve an sinuses. Contrast CT revealed lymphadenopathy of right axillary and few subcentimeter lymph nodes of the neck.

FNAC of cervical and axillary lymph node, chest radiograph, and CT of chest and abdomen were done to know the metastatic extent. FNAC revealed metastasis of malignant melanoma to right cervical and axillary lymph nodes. Chest radiograph, abdominal sonography, and CT of chest and abdomen did not show any significant metastatic lesions.

Since the lesion was large with metastatic extension the patient was referred to higher oncology center for further treatment.

## 3. Discussion

Malignant melanoma was first described by Weber in 1859. It was recognized as a distinct clinical entity and named as “melanotic sarcoma” by Lucke in 1869 [2]. It represents 0.2 to 8% of the total cases of melanoma from the other localizations of the body and 0.5% of all oral neoplasia [1, 6–18]. It occurs between 30 and 90 years of age, with higher incidence in the sixth decade and rarely seen below 20 years of age [19]. Oral melanomas show slightly more male predilection (2 : 1) [1, 13, 14, 19]. And incidence is more common in whites than blacks [6, 10]. Commonly involved intraoral sites are hard palate (32%), maxillary gingiva (16%), mandibular gingiva (7%), tongue (7%), buccal mucosa (7%), and upper and lower lip (7%) [6, 9, 10, 13, 19].

The etiology of oral melanoma is unknown. Exposure to sunlight, denture irritation, chewing tobacco with betel nut, and smoking have been implicated as etiologic factors in the past. However, there has been no evidence to support these theories [1, 6]. Currently, like their cutaneous counterparts, primary oral melanomas are believed to arise either from nevus, preexisting pigmented areas, or* de novo *(30% cases) [1, 4, 7].

Three criteria have been proposed by Gupta et al. that can be helpful in the diagnosis of primary oral melanoma. These include presence of malignant melanoma in the oral mucosa; the exclusion of melanoma at any other primary site; and histopathologic observation of “junctional activity” which is described as melanocytes arranged along the basal layer of the surface epithelium 1 [4, 7, 8, 10, 12]. 

*The Clinical Characteristics: ABCDE Rule of Melanomas.* Asymmetry, in which one half of the lesion does not match the other, borders show irregularity, with blurred, notched or ragged edges, color will be irregular, with non-uniform pigmentation, including brown, black, tan, red, white or blue, diameter will be greater than 6 mm, growth itself being a sign, and elevation, a raised surface also being a sign. Unfortunately, these symptoms appear relatively late in the course of the disease, by which time significant vertical invasion of the tumor cells into the underlying tissues has already occurred [6, 7, 15].

Padhye and D'Souza classified oral melanomas into five types, based on the clinical appearance: pigmented nodular, nonpigmented nodular, pigmented macular, pigmented mixed, and nonmixed [3, 6, 15]. Unpredictable and widespread metastasis is a well-known feature of malignant melanoma. Metastases to regional lymph nodes and distant spread to bone are encountered in end-stage patients. Lymph nodes, central nervous system, lungs, and liver are also common regions for metastasis [7].

Differential diagnosis includes oral melanotic macule, smoking-associated melanosis, medication-induced melanosis (antimalarial drugs and minocycline), melanoplakia, pituitary-based Cushing's syndrome, postinflammatory pigmentation, melanoacanthoma, melanocytic nevi of the oral mucosa, blue nevi, nevi of Spitz, Addison's disease, Peutz-Jeghers syndrome, amalgam tattoo, Kaposi's sarcoma, physiologic pigmentation, pigmentation related to the use of heavy metals, and many other conditions sharing some macroscopic characteristics [2, 4, 7, 20].

Presence of histological features of atypical melanocytes, usually larger than the normal melanocytes and having varying degree of nuclear pleomorphism and hyperchromatism in the epithelial and connective tissue junction in the biopsy of the melanotic lesions of the oral mucosa, is suspicious for oral malignant melanoma [16].

A histological melanoma is confirmed by the Fontana-Masson silver stain and the appropriate immunohistochemical staining pattern that includes HMB-45 antibodies, Melan-A, tyrosinase, and antimicrophthalmia transcription factor. S-100 protein is always positive in melanomas [1, 4, 7].

The treatment policy for oral malignant melanoma is unclear and many authors think it is left to the discretion of the surgeon [8, 18]. However surgery along with chemotherapy, radiotherapy, and immunotherapy remains the most effective treatment for malignant melanoma [5, 14, 15, 17, 18, 20].

The prognosis of patients with oral melanoma is very poor. The reported 5-year survival rate for oral malignant melanoma has ranged from 4.5% to 29% with the median survival rate of 18.5 months after initial diagnosis. The median survival is affected by whether there is lymph node involvement (18 months) or not (46 months) [7, 8].

In the case reported here, the black tinted growth was overlooked and minor surgical procedure was conducted and later growth turned to a massive lesion with regional lymph nodal metastasis. However due to large size and regional lymph node metastasis, prognosis and survival rate of the patient might reduce.

## 4. Conclusion

Most oral malignant melanomas are asymptomatic and painless in early stages, and unfortunately, diagnosis is delayed until symptoms such as swelling, ulceration, and bleeding occur. As an early diagnosis is very important for better survival and prognosis in oral melanoma, thus, early detection of oral melanoma is very critical, whose goal will be achieved by self-examination training and early detection of suspected melanotic lesions by dentist and physicians.

## References

[1] Kumar A., Bindal R., Shetty D. C., Singh H. P. (2012). Primary oral malignant melanoma: clinicopathological series of four cases. *Dental Research Journal*.

[2] Rimal J., Kasturi D. P., Sumanth K. N., Ongole R., Shrestha A. (2009). Intra-oral amelanotic malignant melanoma: report of a case and review of literature. *Journal of Nepal Dental Association*.

[3] Padhye A., D'Souza J. (2011). Oral malignant melanoma: a silent killer. *Journal of Indian Society of Periodontology*.

[4] Gupta M., Gupta M., Aggarwal A., Ahuja R., Pachauri A., Kumar P. (2013). Significance of early detection of oral malignant melanoma: some reasonable facts. *Clinical Cancer Investigation Journal*.

[5] Dimitrakopoulos I., Lazaridis N., Skordalaki A. (1998). Primary malignant melanoma of the oral cavity. Report of an unusual case. *Australian Dental Journal*.

[6] Devi P., Bhovi T., Jayaram R. R., Walia C., Singh S. (2011). Malignant melanoma of the oral cavity showing satellitism. *Journal of Oral Science*.

[7] Pour M. S. H. (2007). Malignant melanoma of the oral cavity. *Journal of Dentistry of Tehran University of Medical Sciences*.

[8] Kumar K., Santhosh B. S., Priya N. K. (2011). Primary oral malignant melanoma—a case report. *Nigerian Dental Journal*.

[9] Chiu T.-T., Lin H.-C., Su C.-Y., Huang C.-C. (2002). Primary malignant melanoma of the tongue. *Chang Gung Medical Journal*.

[10] Ahmadi-Motamayel F., Falsafi P., Baghaei F. (2013). Report of a rare and aggressive case of oral malignant melanoma. *Oral and Maxillofacial Surgery*.

[11] Samaila M. O. (2013). Extra-cutaneous palatal malignant melanoma in middle-aged females. *European Journal of General Medicine*.

[12] Biradar V., Latturiya R., Biradar S. (2012). Late diagnosis of oral mucosal melanoma: case report. *Journal of Dental & Allied Sciences*.

[13] Guevara-Canales J.-O., Gutiérrez-Morales M.-M., Sacsaquispe-Contreras S.-J., Sánchez-Lihón J., Morales-Vadillo R. (2012). Malignant melanoma of the oral cavity. Review of the literature and experience in a Peruvian population. *Medicina Oral Patologia Oral y Cirugia Bucal*.

[14] Lessa N. L., Moleri A. B., Merly F., Moreira L. C., Moreira M. J., Antunes H. S. (2008). Oral melanoma: an unusual presentation. *Dermatology Online Journal*.

[15] Sharma N. (2012). Primary oral malignant melanoma: two case reports and review of literature. *Case Reports in Dentistry*.

[16] Shreedhar B., Sharma D., Chaturvedi M. (2012). Oral malignant melanoma: a case report. *International Journal of Dental Clinics*.

[17] Khalifa H., Abdullah S., Sallam K., Khalil H., Moneim I. A., Elaffandi A. (2009). Primary malignant melanoma of the tongue. *Canadian Journal of Surgery*.

[18] Gondivkar S. M., Indurkar A., Degwekar S., Bhowate R. (2009). Primary oral malignant melanoma—a case report and review of the literature. *Quintessence International*.

[19] Goel A., Sreenivasan V., Patil P., Juneja N. (2013). Oral malignant melanoma—a review. *International Dental Journal of Student’s Research*.

[20] Ullah H., Vahiker S., Singh M., Baig M. (2010). Primary malignant mucosal melanoma of oral cavity—a case report. *Egyptian Journal of Ear, Nose, Throat and Allied Sciences*.

